# Management of plant species for controlling pests, by peasant farmers at Lagoa Seca, Paraíba state, Brazil: an ethnoecological approach

**DOI:** 10.1186/1746-4269-2-42

**Published:** 2006-10-06

**Authors:** Andréia de Souza Guimarães, José da Silva Mourão

**Affiliations:** 1 Universidade Federal da Paraíba, Centro de Ciências Agrárias, Departamento de Fitotecnia, Programa de Pós-Graduação em Agronomia. Areia-PB, Brazil; 2 Universidade Estadual da Paraíba, Centro de Ciências Biológicas e da Saúde, Departamento de Biologia, CEP: 58109-753- Campina Grande-PB, Núcleo de Etnoecologia, Educação Ambiental e Gestão, Brazil

## Abstract

Ethnoecological knowledge may be understood as spontaneous knowledge, culturally referenced of any society's members, learned and transmitted through social interactions and that are targeted at resolution of daily routine situations. The traditional knowledge in small scale economy societies as well as the non-academic knowledge in urban-industrial societies might be included in this concept. An ethnoecological approach study was performed here on people living at the communities of Alvinho, Almeida, Chã do Marinho, Floriano, and Chã de Oiti, all located in the municipality of Lagoa Seca, Paraíba state, Northeast Brazil. The general objective pursued here was to study the knowledge that peasant farmers have on management of plant species utilized for pest control. For this, the methodological instruments employed here to investigate the ethnoecological knowledge were: direct observation, structured and semi-structured interviews, and tours conducted by local peasant farmers. We analyzed the data obtained under an emic/etic view and also by comparing the local knowledge with those obtained from the literature. The results showed that people in those communities utilize management alternatives for controlling pests, which are mainly: (i) fallowing; (ii) crop rotation; (iii) destruction of crop remains and fruits attacked by pests; (iv) alternations of crops with repellent plants; and/or (v) mixed cropping; (vi) insect's larvae covered with soil; (vii) crops irrigated abundantly; and (viii) soil preparation. The recovery and comprehension we get about this knowledge as well as the farmers' *savoir faire*, are extremely important to the revival of ancient agricultural practices, which have been forgotten due to advances in modern agriculture. The data obtained here showed that a huge body of knowledge the farmers have on many forms or strategies of management are generally compatible with scientific knowledge.

## Background

The systematic knowledge of crops management for pest control is not an invention of modern western science. Many communities of peasant farmers and indigenous peoples developed their own strategies by using natural chemicals against crop pests. Such system results from people's experience throughout generations in interaction with their environments, since externally financial resources and scientific knowledge have not been accessible to them [1]. Studies on popular wisdom concerning the knowledge that local human populations have about nature are extremely important for the valuation of people's traditional ecological knowledge itself, aiming the appropriate management of natural resources. As formerly stated [2] "the administration or management of natural resources aim to use the ecosystems in a way their perpetuity and degree of utilization are maintained environmentally sustainable"; these would improve the traditional peoples' life conditions and the conservation of their natural and cultural inheritance.

All different forms of view, representation and practices of pest control have been discussed in the present study through a theoretical methodological focus provided by ethnoecology, as reported elsewhere [[3-5] and [6]]. Ethnoecological research in general, is based on a view that conservation of nature is associated directly to social, economical, cultural, and biological issues. Ethnoecology [6] presupposes the investigation of human populations' knowledge about nature, based on peoples' beliefs, their traditional knowledge, perceptions, and management of natural resources. Such approach will certainly contribute for implementing management programmes, which not only take in account natural resources themselves, but also the human populations that live on them. Such programmes might be useful for an ecological and socially responsible policy or yet, as guarantee for the preservation of the traditional *savoir faire*, which makes up the human cultural universal heritage. The interdisciplinary nature of ethnoecology is another relevant aspect that promotes articulation and integration between formal knowledge (researchers' experience on pest control shared among them) and local people's knowledge (rural or indigenous peoples).

In this way, it was aimed in the present study to describe the knowledge and practices utilized by traditional peasant farmers of the municipality of Lagoa Seca, Paraíba state, for managing crops pest control.

## Methodology

The study area

The municipality of Lagoa Seca is located 129 km of João Pessoa. It makes part of the 'agreste' and 'brejo' (traditional microregions or zones between the Paraiban littoral and the semi-arid 'sertão', a western microregion), at the eastern side of the Planalto da Borborema (the Borborema Upland). It is located at an altitude of 634 m, covering an area of 133 km^2 ^[7]. The geographic coordinates of Lagoa Seca are between lat 7°10'15"S and long 35°51'13"W, limited on the north by the municipalities of Montadas, São Sebastião de Lagoa de Roça, and Areial; on the west by Puxinanã; on the south by Campina Grande; and on the east by Massaranduba [7].

The target populations of the present research make part of the communities of Alvinho, Almeida, Chã do Marinho, Floriano, and Chã do Oiti, where peasant farmers carry out agricultural activities, cultivating mainly maize, bean, and green vegetables. They are categorized as farming family's activity involved in traditional subsistence agriculture. Some authors [8,9] consider the family as the core of all families' activity, where as well as producing and consuming, the family's strategies must satisfy the needs of the domestic group and the exploitation unit's demands, at the time they also get the means to satisfy the social or institutional requirements of the productive unit.

## Methodological procedure

Our field work was carried out in the studied communities by selecting the peasant farmers according to criteria [10] which take into account the affinity of their habits with the community, and also with the easy access to the area where they live and apply traditional agricultural techniques. The study period was from July to October 2002, with weekly visits to the areas. The data were initially obtained in informal talks, casual meetings and open or free interviews with *ca*. 50 farmers from the communities. On the basis of identified themes, the methodological techniques employed here were free interviews, direct observation, and application of structured and semi-structured questionnaires to farmers with considerable expertise. These farmers were suggested by other communities' members, and their number varied according to the subject-matter. Such technique has been denominated as 'snow ball', where the own members of the communities suggest their fellows to be interviewed [11].

The data obtained were transcribed and codified on the basis of themes and identified emic categories and they were organized in a data bank. We chose the option of an essentially qualitative approach and speech analysis, with quantitative approach only in specific situations. The results were analyzed through the union model of several individual competences [12]. Direct observation was applied as a way to get better understanding and description of agricultural techniques they use. We also counted with the help of guided tours, as recommended elsewhere [13]. Specimens of pests that attack their crops were collected with their help, during their crop management work. We recorded the vernacular names they mentioned, being later confirmed by elderly farmers. Researchers at the EMBRAPA – Empresa Brasileira de Pesquisa Agropecuária, in the national centre for research on cotton crop, at Campina Grande, state of Paraíba, identified the species we collected.

## Results and discussion

The results obtained here show interaction of factors in farmers' management of plants as they prepare and fertilize the soil that is hoed into ridges, being crops planted on them, irrigated and later their produces are harvested. This process is subsequently adopted by each family and so the soil is maintained potentially productive. This cropping system, up to the harvest, may suffer interferences interrupting the production cycle, like crop pests to which the farmers put a high valuation owing to their hazardous effect on production.

This way, the peasant farmers were stimulated to find out several forms or strategies of management, through practical knowledge, so preventing the development of some pests in that region.

The interactions of soil, air, water and plants, are clearly and permanently necessary to the communities' life, being then quite important to keep these factors in equilibrium by means of a simple and healthy management. According to the farmers studied here, management means the ability of the use and re-use of a certain area, by preventing its exhaustion. The concept of natural resources emerged intensively from the 1980s when natural and social sciences researchers insisted on demonstrating the relationship of environmental degradation and questions of social justice, impoverished rural population, and Indians rights [14]. These latter authors stated that such concept has included the practices of participative management spread over the Amazonas in the past century. Since those times several family groups of producers got involved in managerial initiatives at their communities, in response to pressures people exerted in common on the resources they have always depended on for living.

## Management techniques of cultivated crop species

The data obtained in this work showed that the farmers use eight techniques for managing their cropped plants, namely: (i) fallowing; (ii) crop rotation; (iii) destruction of crop remains and fruits attacked by pests; (iv) alternations of crops with repellent plants; and/or (v) mixed cropping; (vi) insect's larvae covered with soil; (vii) crops irrigated abundantly; and (viii) soil preparation. The technique they apply to control the most common crop pests, constitutes also an important aspect of their knowledge. Similar management techniques were also studied by other authors [15,16], where the following stages of the farmers' agricultural process were reported as important, *e.g*. fertilization, use of water, soil maintenance, application of biocides, crop rotation, and interplanting with repellent plants. Those authors pointed out that some of these stages are beneficial to increase nitrogen content, reduction of soil erosion, nutrients turnover, and increase of productivity.

Information on types of management and forms of control of the main pests in the study area are shown in Table 1 (see Table 1). It can be observed that crop rotation is predominant in horticultures, which are planted and harvested in short periods of time and are greatly susceptible to pests.

**Table 1:** Peasant farmers' knowledge on forms or strategies to control pests, the procedure they adopt and the pests that are susceptible to treatment, in the municipality of Lagoa Seca, state of Paraíba.

**Types of management**	**Procedure**	**Pests (susceptible to treatment)**
Crop rotation	Rotated crops: lettuce, *Lactuca sativa*; coriander, *Coriandrum sativum*; scallion, *Allium fistulosum*; carrot, *Daucus carota*; cabbage, *Brassica oleracea*	'Cachorrinho-d'água' (an orthopteran, a kind of mole cricket that attacks plant roots); an unidentified little caterpillar; 'lagarta-de-rosca' (a noctuid moth, *Agrotis ipsilon*, living at the base of host plants or in the soil)
Destruction of fruits and crop remains	Burning of fruits (slightly attacked fruits); removal of crops followed by burning (fruits strongly attacked)	'Mosca branca' (white fly, a dipteran, *Bemisia argentifoli*); 'mosca-das-frutas' (a fruit fly, *Ceratitis capitata*); an unidentified caterpillar
Mixed cropping, with repellent plants	Cravo-de-defunto (a marigold, *Tagetes minuta*)	'Pulgão-do-algodoeiro' (a kind of cotton plant louse, *Aphis gossipy*); unidentified species of caterpillar, mite, and moth
Irrigation water in abundance on crops	Jets of water, by using host, are shot on pest's patches on the crop	Unidentified species of caterpillars and ants, in general
Caterpillars covered with soil	A handful of soil is put on pest's patches on the crop	Caterpillars of *Helicoverta *sp., that attack corncobs and of *Spodoptera frugiperda*, that attack the maize plant
Soil preparation	Organic amendments are prepared and fallowed from 8 to 22 days, being applied lately twice during winter (the rainy season)	Applied on the pests above-mentioned

## Description of techniques

One technique the farmers generally use is fallowing, where according to them the land needs time to recover for the next planting. This is an entirely valid reason for a profitable and healthy harvest, because well-regenerated and fertile soil is quite important for the prevention of pest attacks, as they say:

'*A soil must be put at rest for producing good fruits*'.

In studies performed on fishermen at the lower course of the River São Francisco, in the state of Alagoas [3], the author reported that soil could be utilized when nutritionally rich ('strong soil') or not ('feeble soil'). In this latter condition they plant pineapple, while in the former, polyculture is usually practised. Thus, a 'feeble soil' is not useless but it is used for restricted crop yield.

Studies performed on fallowing among Kayapó Indians for longer than three months [17] revealed the effectiveness of this system for controlling harmful pests. In similar studies on Kuikura Indians' farming practised on cleared grounds prepared for planting, at the higher course of River Xingu [18], the author observed that natural biological control applied by those natives was a quite important strategy that deserves further studies on its use.

Crop rotation is reported here as a very common strategy applied by the peasant farmers in our study area, for attenuating pest attacks in their crops. They reason they avoid monocultures because of their greater susceptibility to pests, which would put the crops at risk.

A crop rotation practised by the farmer may inhibit development of a pest throughout the year by reducing its population and consequently will cause lesser damage in the subsequent plantings. Kayapó Indians in their agricultural activities showed a revival of old practices and management when using appropriately the environment for controlling pests [19]; they used crop rotation and fallowing for minimizing pest attacks.

Crop rotation is an annual alternation of different plant species in the same plantation area. The species the farmers select for cropping are intended to generate commercial profit and recovery of soil productive properties. At the time the farmers obtain a diversified production of food and other produces, if the crop is appropriately managed for a quite reasonably long period, some benefits will certainly be achieved, as follows: (i) improvement of physicochemical and biological properties of the soil; (ii) it helps to control weeds, diseases, and pests; and (iii) it renews the soil organic matter, which will protect the soil against the action of climate agents. Additionally, direct drilling would be a viable alternative to be applied and would certainly benefit farming and environment as a whole [20].

In a study on horticulturists of northern littoral of Natal, state of Rio Grande do Norte [20], it was reported that local farmers practise crop rotation, resulting in higher crop resistance to pests. Mixed cropping and/or crop rotation was reported to avoid catastrophic attacks of insects and pests, and plant cover might suppress effectively the growth of weeds and the need to control them might be reduced.

The other technique here investigated was the destruction of fruits attacked by pests or diseases. Such fruits are collected and burned to eliminate the pest and avoid its dissemination and high populations in subsequent crops. The farmers state that when there is a low level of attack, the fruits are their main target of destruction otherwise the entire crop will be destroyed. Crop remains are also destroyed as a complement to this technique. This sort of management avoids new incidence of the pest in the subsequent crop, as observed by a local farmer:

'*We have to burn, or the pest will spread out*'.

In the community studied here, the farmers themselves observed that mixing a crop with repellent plants, like a species of marigold (*Tagetes minuta*), will make the pest to recede owing to the flower's smell.

It was reported from laboratory experiments [21] that extracts obtained from *T. minuta *caused a high control index of *Sitophilus *spp., higher than 85%, deemed as of high toxicity, being possible its use as an alternative insecticide. Later [21], also in laboratory experiments, extract from *T. minuta *caused 100% mortality to termites (Termitidae, Nasutitermitinae). Mixed crop, made up of pumpkin plus maize plus plants (tomato and marigold) that act as repellent of pests, have been proposed for use to avoid pests [22].

A woman-farmer commented about such technique:

'*One day I thoughtfully said: I'm going to find out a way to kill those little animals because the flowers's smell is quite strong; then I started by rubbing the marigold's flower on plant's parts that had those little animals and they were all killed*'.

The use of jets of water applied on plants infected by pests is another management technique that kills the pests or carries them away, and their incidence is consequently reduced. Actually, it has been observed [23] that abundant water applied on crops reduces pests because they are removed from their infestation sites or are drowned; and sometimes, entomopathogenic organisms attack the pests under high humidity conditions.

Some crops attacked by the caterpillar of *Spodoptera frugiperda*, at initial phase of infestation, are combated by covering the larvae with *ca*. 100 g of soil, mainly on the ones situated between the leaves of maize which will die after some minutes. This procedure is utilized by the farmers at the beginning of the insect attack, preferably during summer, as the high temperature will kill them more quickly.

Daniel Duarte -UFPB (personal communication) suggests that a soil fungus may act as an entomopathogen of the insect, killing it rapidly. But one of the peasant farmers gives this explanation:

'*Just a little bit of soil we put on the caterpillars make them die of suffocation; but only if they are not many*'.

Soil preparation technique involves the use of manure from cattle, goats, and chickens, which the farmers let to lie fallow for three days. Then they are applied in small amount because of their high combustion potential.

What makes us to believe that the peasant farmers know the importance of preparing the soil for sowing the seeds, their sprouting, and unlimited crop yield, is confirmed from the following statement of one of them:

'*The plant is like us; if we're weak, it'll be easier to get ill*'.

The practical results of the techniques above-described are the produces harvested in the right time, and after land fallow, new seeds can be planted. Harvest is carried out from alternate ridges that the farmers construct on steep slopes, forming terraces. The ridging pattern acts as a set of barriers to the down slope movement of water and promotes infiltration, a fact the peasant farmers know for obtaining a dynamical crop yield. This procedure when associated to the other techniques described here, have been used by them because they know these are essential measures to guarantee pest control and high crop yield.

## Conclusion

Most of the crop management techniques used by the peasant farmers studied here stem from their '*savoir faire*' they experience in their daily life in the field. The most practised management strategies of the farmers are crop rotation and land fallow, which are practical and effective. The other techniques are less used. Their knowledge on crop management varieties without application of toxic chemicals showed to us the '*corpus*' and '*praxis*' importance for controlling pests, when compared to scientific knowledge. For this reason, we understand that their knowledge must be at issue when pro-conservation attitudes are under discussion.
